# Metabolic Deregulations in Patients with Polycystic Ovary Syndrome

**DOI:** 10.3390/metabo13020302

**Published:** 2023-02-17

**Authors:** Marzena Jabczyk, Justyna Nowak, Paweł Jagielski, Bartosz Hudzik, Karolina Kulik-Kupka, Aleksander Włodarczyk, Katarzyna Lar, Barbara Zubelewicz-Szkodzińska

**Affiliations:** 1 Department of Metabolic Disease Prevention, Faculty of Health Sciences in Bytom, Medical University of Silesia, 41-900 Bytom, Poland; 2 Department of Nutrition and Drug Research, Institute of Public Health, Faculty of Health Sciences, Jagiellonian University Medical College, 31-066 Cracow, Poland; 3 Third Department of Cardiology, Silesian Center for Heart Disease, Faculty of Medical Sciences in Zabrze, Medical University of Silesia, 41-800 Zabrze, Poland; 4 District Hospital, 41-940 Piekary Slaskie, Poland; 5 Department of Endocrinology, District Hospital, 41-940 Piekary Slaskie, Poland

**Keywords:** polycystic ovary syndrome, atherogenic indices, anthropometric indices, metabolic disorder

## Abstract

Polycystic ovary syndrome (PCOS) contributes to endocrine and metabolic complications for women worldwide. The aim of this study was to establish the usefulness of new anthropometric indices and atherogenic indices in the evaluation of metabolic disorders, in particular, glucose and insulin abnormalities in the profiles of women with polycystic ovary syndrome (PCOS). In the study, a total of 49 women with PCOS aged between 18 and 39 years were recruited. All patients were tested for fasting glucose and insulin, lipid parameters, oral-glucose administration, and biochemical parameters. All of them underwent anthropometric measurements, such as BMI (body mass index), WHR (waist-to-hip ratio), WHtR (waist-to-height ratio), BAI (body adiposity index), VAI (visceral adiposity index), LAP (lipid accumulation product), BRI (body roundness index), ABSI (A body shape index), AIP (atherogenic risk of plasma), AC (atherogenic coefficient), Castelli risk index-I, Castelli risk index-II and (LCI) lipoprotein combine index, TG/HDL-C ratio, METS-IR (The metabolic score of insulin resistance), triglyceride glucose index (TyG index), triglyceride glucose-body mass index (TyG-BMI index) and triglyceride glucose-waist circumference index (TyG-WC index) were calculated. The analyzed anthropometric measurements/indices and atherogenic indices demonstrated significant correlations in PCOS women. T A strong relationship was found between fasting glucose, fasting insulin, glucose after 60 min, HOMA-IR index in the patients with PCOS. There was no significant relationship between HbA1c and other analyzed parameters and indices. Most of the analyzed anthropometric and atherogenic indices may be useful tools in evaluating metabolic disorders, and, in particular, glucose and insulin abnormalities in PCOS women.

## 1. Introduction

Polycystic ovary syndrome (PCOS) is a complex endocrine disorder affecting between 6% to 22% women worldwide [1]. It is characterized by ovulatory dysfunction, hyperandrogenism, and polycystic ovarian morphology [1,2]. It leads to numerous conditions, including, but not limited to: dyslipidemia, hyperinsulinemia, insulin resistance (IR), impaired glucose metabolism, type 2 diabetes mellitus (T2DM), metabolic syndrome (MetS), hyperandrogenism, oxidative stress, and infertility [1,3,4,5]. The pathogenesis of PCOS is complex and the etiology still requires further examination. Numerous studies have explored the mechanisms of metabolic dysregulation (glucose and lipids) as well as inflammatory status in the pathogenesis of PCOS. IR is now a well-recognized feature of PCOS and, in association with hypertension and dyslipidemia, may increase the risk of cardiovascular (CV) and cerebrovascular events. These risk factors are intensified by central obesity, which is present in the majority of women with PCOS [6]. Metabolic dysregulation puts PCOS women at higher risk of developing CV disease (CVD) [1]. Adolescent girls with obesity and PCOS were reported to have elevated fasting and postprandial plasma triglycerides and ApoB-lipoprotein remnants levels [7]. This could be a marker of early subclinical CVD risk. Additionally, many studies have demonstrated that PCOS women have significantly higher levels of the triglyceride-to-high density lipoprotein cholesterol (TG-HDL-C) ratio, IR, and cardiometabolic risk factors. Lipid indices have been highly associated with impaired insulin metabolism and hyperandrogenemia [8,9,10]. Zheng et al. concluded that new metabolic lipid indices (TyG index-triglyceride glucose index; TyG-BMI index-triglyceride glucose-body mass index; TyG-WC index-triglyceride glucose-waist circumference index) seem to be useful in early identification of the risk of prediabetes and diabetes, which are also common in PCOS women [11]. Considering that adipose tissues secrete adipokines, inflammatory cytokines, reactive oxygen species, and may lead to a variety of metabolic disorders, such indices may be even better predictors of IR than TyG-index alone [12]. Additionally, a novel non-insulin-base score METS-IR (which combines anthropometric measurements and non-insulin fasting laboratory values) could be useful in evaluating insulin sensitivity and detecting IR in patients at risk of developing T2DM [13]. Therefore, METS-IR as well as the lipoprotein combine index (LCI)-a new risk predictor for coronary artery disease (CAD) are promising scores to evaluate cardiometabolic risk in women PCOS [14,15].

Adiposity plays a significant role in maintaining and moderating PCOS. The anthropometric indices such as: waist-to-hip ratio (WHR), waist-to-height ratio (WHtR), visceral adiposity index (VAI), body adiposity index (BAI), lipid accumulation product (LAP), body roundness index (BRI), ABSI A body shape index (ABSI) could also be markers of adipose tissue abnormalities and CVD risk at PCOS [16,17].

The aim of this study was to investigate the usefulness of new anthropometric indices (BAI) body adiposity index; (VAI) visceral adiposity index; (LAP) lipid accumulation product; (BRI) body roundness index; (ABSI) A body shape index, and new atherogenic indices (Castelli index-I; Castelli index-II; (AIP) atherogenic risk of plasma; (AC) atherogenic coefficient; (LCI) lipoprotein combine index; (TG/HDL-C ratio) ratio of triglycerides to HDL-Cholesterol; (METS-IR) the metabolic score of insulin resistance; (TyG index) triglyceride glucose index; (TyG-BMI index) triglyceride glucose-body mass index; (TyG-WC index) triglyceride glucose-waist circumference index) in the evaluation of metabolic disorders, in particular glucose and insulin profiles, in patients with PCOS.

## 2. Methods

### 2.1. Study Population

The study enrolled 49 women. Inclusion criteria were: PCOS diagnosis based on the 2003 Rotterdam criteria (at least two out of three criteria listed below were met: oligo-ovulation or anovulation, clinical and/or biochemical hyperandrogenism, polycystic ovaries visualized on ultrasound–12 or more follicles of 2–9 mm in diameter in each ovary and/or increased ovary volume > 10 mL [18].

Exclusion criteria were: the patient’s lack of consent to participate in the study; pregnancy; no blood collected as part of routine or hemolysis of the blood; use of hormonal contraceptives, glucocorticosteroids, oral steroid medications or lipid-lowering drugs, drugs that affect glucose metabolism; previously diagnosed and treated diabetes mellitus; decompensated thyroid disorders; androgen excess disorders (congenital or late-onset congenital adrenal hyperplasia, hyperprolactinemia, Cushing’s disease/syndrome, androgen-secreting tumors, idiopathic hirsutism); depressive disorders and treatment of depression; diagnostic incomplete; re-hospitalization.

The study conforms to the Declaration of Helsinki and was reviewed and approved by the local bioethics committee (KNW/0022/KB1/143/15). Informed consent for data analysis was obtained from all participants.

### 2.2. Methods and Laboratory Measurements

The study was conducted from 2015 to 2018 in the Department of Endocrinology, Piekary Medical Centre, St. Luke’s Local Hospital in Piekary Śląskie, Poland.

Blood samples were taken from patients in the morning, before breakfast, and 1 mL of blood collected as part of routine testing was preserved for further analysis, which after centrifugation was frozen and stored at −70 degrees Celsius.

The value of the HOMA-IR (Homeostatic model assessment) index was calculated using the following formula:

HOMA-IR = fasting insulinemia (mU/mL) × fasting glycemia (mmol/L)/22.5 [19].

The value of the METS-IR (The metabolic score for insulin resistance) was calculates as (In ((2 × fasting glucose) (mg/dL) + TG (mg/dL) × BMI)/(Ln (HDL-C) (mg/dL)) [14].

TyG index was computed using the formula following: In [fasting glucose (mg/dL) × TG (mg/dL)/2] [19].

TyG-BMI index was defined as: [fasting plasma glucose (mg/dL) × fasting triglicerydes (mg/dL)/2] × BMI [20].

TyG-WC was defined as: [fasting glucose (mg/dL) × TG (mg/dL)/2 × WC] [20].

LCI was calculated using the following formula: ((TC (mmol/L) × TG (mmol/L) × LDL-C (mmol/L))/HDL-C (mmol/L)) [15].

Castelli’s risk index-I was calculated according to the following formula [9] = (TC/HDL-C).

Castelli’s risk index-II was calculated according to the following formula [9] = (LDL-C/HDL-C).

Atherogenic coefficient (AC) was calculated according to the following formula [9] = (TC-HDL-C/HDL-C).

Atherogenic index of plasma (AIP) was calculated according to the following formula [9] = (log(TG/HDL-C)).

Trigliceryde to HDL-cholesterol was calculated according to the following formula [9] = (TG/HDL-C).

Anthropometric parameters were measured with the use of standard methods in the morning. These measurements included body weight [kg], height [cm], waist circumference [cm], and hip circumference [cm].

BMI was calculated according to the following formula [21]:

BMI = body weight [kg]/height [m]^2^;

WHR was calculated according to the following formula [22]:

WHR = waist circumference [cm]/hip circumference [cm];

WhtR was calculated according to the following formula [22]:

WHtR = waist circumference [cm]/height [cm];

BAI was calculated according to the following formula [23]:

BAI = (hip circumference [cm]/height [m]^1.5^) [18];

VAI was calculated according to the following formula [19]:

VAI = [waist circumference [cm]/(36.58 + (1.89 × BMI))] × (triglyceride concentration [mmol/L]/0.81) × (1.52/HDL concentration [mmol/L]);

LAP was calculated according to the following formula [19]:

LAP = (waist circumference [cm] − 58) × (triglyceride concentration [mmol/L]);

BRI was calculated according to the following formula [24]:

BRI = 365.2 − 365.5 × √(1 − (((WC/2π)2)/[(0.5 × height)]^2^);

ABSI was calculated according to the following formula [24]:

ABSI = WC[m]/[(BMI)^2/3^) × (height [m])^1/2^)].

### 2.3. Statistical Analysis

Statistical analysis was performed using the STATISTICA 13 PL software (Tulsa, Oklahoma, OK, USA). The Shapiro–Wilk test was used to test the distribution. Continuous variables were expressed as means ± standard deviations (normal distribution) or median and interquartile range (distribution other than normal). Normally distributed data were compared using the Student’s t-test, while nonparametric data were compared using the Mann–Whitney U test. A correlation between variables was evaluated using the Pearson’s correlation coefficient (normal distribution) and the Spearman’s rank correlation coefficient (non-normal distribution). A *p* value of less than 0.05 was considered significant.

## 3. Results

### Characteristic of the Study Group

The study included 49 women with a PCOS diagnosis. The median age of the women was 25 (22–29) years. Baseline clinical and laboratory characteristic as well as anthropometric parameters of the study group are shown in Table 1.

Correlations between anthropometric parameters, atherogenic indices and glucose profile in the study group are shown in Table 2.

There was an observed positive correlation between fasting glucose (mmol/L) and analyzed parameters (body weight, waist and hip circumference, BMI, WHR, WHtR, BAI, VAI, LAP, BRI, Castelli’s risk index-I, Castelli’s risk index-II, AC, LCI, METS-IR, TyG index, TyG-BMI index, TyG-WC index) (*p* < 0.05) excluding ABSI and AIP (*p* > 0.05). Glucose after 120 min was observed as positively correlated with analyzed anthropometric parameters such as WC, BMI, WHR, WHtR, VAI, LAP, BRI, as well as atherogenic indices such as Castelli’s risk index-I, Castelli’s risk index-II, AIP, AC, LCI, TG/HDL ratio, METS-IR, TyG index, TyG-BMI index, TyG-WC index (*p* < 0.05).

There was a significant positive correlation between glucose after 60 min and the remaining laboratory results, as well as anthropometric measurements/indices excluding WC and BAI (*p* < 0.05).

Correlations among anthropometric parameters, atherogenic indices and insulin profile in the study group are shown in Table 3.

Significant correlation was found between all analyzed indices and the fasting insulin (mmol/L) (*p* = 0.000). Indeed, insulin after 120 min was observed to be positively correlated to anthropometric parameters such as body weight, waist circumference, BMI, WHR, WHtR, LAP, BRI and atherogenic indices as METS-IR, TyG index, TyG-BMI index, TyG-WC index (Table 3).

The HOMA-IR index was strongly correlated among the group of women to all analyzed anthropometric parameters and atherogenic indices (*p* = 0.000), (Table 4).

No significant correlation was found between HbA1c [%] and analyzed anthropometric measurements and indices among study group (*p* > 0.005).

## 4. Discussion

PCOS is a condition where metabolic disorders are estimated to coexist in 75% of women with PCOS of a proper weight, and in 90% of obese women with PCOS. Fifty percent of them progress to metabolic syndrome, which refers to impaired glucose tolerance, hypertension, dyslipidemia, central adiposity, and CVD [25]. Insulin resistance contributes to CVD among other metabolic disorders. Studies have highlighted that IR can be a useful index to determine the PCOS phenotype [26]. On the other hand, hyperinsulinemia and IR are associated with PCOS pathogenesis, and it is not a criterion in the definition of PCOS [27]. However, some disturbances in insulin signaling and steroid hormones have been known to underly the pathophysiology of PCOS [4]. Metformin is one of the possible medical management tools to improve metabolic condition through decreasing insulin resistance in women with PCOS [28].

Women with PCOS and additional risk factors, such as insulin resistance and/or hyperandrogenism, are at particular risk for cardiovascular complications. PCOS should be considered an important CVD risk factor that is associated with insulin resistance, which occurs independently of obesity. [25]. Hiperinsulinemia exacerbates the hyperandrogenism. Due to hyperglycemia and hyperinsulinemia, the hepatic sex hormone binding globulin (SHBG) decreases, which exacerbates the effect of luteinizing hormone and excessive androgen production. Moreover, the administration of insulin in type 1 diabetes may elevate ovarian insulin exposure, leading to excessive synthesis of androgen. The insulin drives production of adipose tissue testosterone [28]. In our study we focused on analyzing the glycemic parameters in PCOS women.

Nawrocka et al. found that 27.6% women with PCOS had insulin resistance, and that hyperandrogenism was found in 48.3% of the studied women [25]. Nagshband et al. reported the prevalence of metabolic syndrome in 59.3% of Indian women with PCOS [16] Abruzzese et al. reported a diagnostic accuracy of LAP in Argentinian young women with PCOS and insulin resistance [26]. Similarly, the accuracy of LAP in demonstration with MetS and glucose intolerance has been reported in Chinese [29], Brazilian [30] and Caucasian [31] women with PCOS.

We demonstrated a strongly relationship with fasting glucose, fasting insulin, and after 60 min and 120 min (only glucose) with the analyzed parameters/indices in PCOS women. Notably, there was no relationship with HbA1c and analyzed parameters/indices in women with PCOS. Similar results have been reported by Cree-Green et al. [32]. However, they have reported the correlation between IR (HOMA-IR R = −0.70, *p* < 0.001) and impaired tolerance of glucose in women with PCOS. However, the review published in Lancet reported that the incidence of type 2 diabetes is higher in women with PCOS [28]. (4.19 per 1000 population per year in a cohort of community-based women with PCOS aged 18–42 years compared to those without PCOS 1.02 per 1000 population [*p* < 0·001]) [33].

In the present study the anthropometric index, ABSI, which is a combination of BMI, WC and height, was found to have less signification than other anthropometric indices. However, BMI and WC alone were significantly correlated with the analyzed parameters (except HbA1C) in the study group of women. Similarly, Naghshband et al., in a cross-sectional study, examined 150 PCOS women and reported that ABSI was not significantly higher in PCOS women with MetS [16]. The authors recommended other anthropometric indices: VAI and LAP for MetS diagnosis in women with PCOS. The simple index of VAI, which indicates the combination of BMI, WC, TG and HDL, is more convenient than using them independently for determining the CVD risk and MetS in PCOS women. Our study showed a significant association of VAI and LAP with metabolic disorders (both glucose/insulin levels and lipid profiles) in PCOS women (*p* = 0.000).

The accuracy of the TyG index has been reported in some populations, particularly in relation to glucose disorders and IR [11,34,35,36]. An Iranian study positively assessed the diagnostic ability of TyG in PCOS women [2]. We have obtained similar results. Ghaffarzad et al. reported a significant association between the lipid parameters TC/HDL-C, TG-HDL-C and LDL-C/HDL-C ratios with IR [37]. Hence, the study was the first to assess the diagnostic ability of TyG among Iranian women with PCOS. Their findings indicated that the lipid profile values were not useful indicators for IR assessment, hence the TyG, TG/HDL-C and TC/HDL-C indices were significantly correlated with IR in the women diagnosed with PCOS.

The assessment of anthropometric and atherogenic indices is a simple, quick, inexpensive, and non-invasive tool that can be used by clinicians and nutritionists in the treatment of women with PCOS syndrome. By determining the above indicators, it is possible to estimate cardiovascular risk, or determine the PCOS phenotype. In addition, assessment of carbohydrate metabolism provides the opportunity to implement appropriate treatment targeting the cause.

The strength of this study is that, to the authors’ current knowledge, it is the first to examine the correlation of multiple anthropometric and atherogenic indices and glucose and insulin profiles in patients with PCOS. The study has also several limitations, which include a small sample size and the inclusion of women from only one geographical region. The criteria for diagnosing glycemic abnormalities in other populations may differ. Further studies should be carried out in other populations to investigate the usefulness of each of the anthropometric and atherogenic indices.

## 5. Conclusions

In summary, this study analyzed new anthropometric indices (BAI, VAI, LAP, BRI) and new atherogenic indices (AIP, AC, Castelli’s risk index-I, Castelli’s risk index-II, METS-IR, TG/HDL-C ratio, TyG index, TyG-BMI index, TyG-WC index), and demonstrated a significant relationship with glucose and insulin profiles in women with PCOS. In consequence, they may be useful in assessing metabolic dyregulations, in particular, with glucose and insulin levels in PCOS women. Further studies are needed to assess the usefulness of metabolic screening and management in patients with PCOS.

## Figures and Tables

**Table 1:** Baseline anthropometric measurements, clinical and laboratory characteristics.

Parameter	Total Group Me (Q1–Q3)
Age (years)	25 (22–29)
Anthropometric measurements	
Body weight (kg)	76.20 (46.30–123.80)
Height (cm)	165.50 (150.50–175.00)
Waist circumference (cm)	88.50 (64.00–132.00)
Hip circumference (cm)	108.00 (86.00–139.00)
BMI index (kg/m^2^)	27.49 (18.03–43.60)
WHR	0.82 (0.63–1.01)
WHtR	0.54 (0.39–0.78)
BAI (%)	33.43 (23.33–45.55)
VAI	1.22 (0.27–6.12)
LAP	36.16 (3.84–170.75)
BRI	4.22 (1.52–10.25)
ABSI	0.08 (0.06–0.09)
Biochemical parameters	
Fasting glucose (mmol/L)	4.94 (3.89–7.60)
Fasting insulin (pmol/L)	96.15 (14.35–299.20)
Glucose after 60 min (glucose tolerance test) (mmol/L)	7.99 (2.66–14.76)
Insulin after 60 min (glucose tolerance test) (pmol/L)	703.15 (173.64–2047.75)
Glucose after 120 min (glucose tolerance test) (mmol/L)	6.47 (4.00–13.76)
HbA1c (%)	5.40 (0.95–5.70)
HOMA-IR index	2.65 (0.35–10.80)
Total cholesterol (mmol/L)	4.55 (3.08–7.45)
HDL cholesterol (mmol/L)	1.66 (0.73–2.86)
LDL cholesterol (mmol/L)	2.41 (1.49–5.14)
Triglycerides (mmol/L)	0.93 (0.44–2.76)
Atherogenic indices	
Castelli’s risk index-I	2.92 (1.83–6.94)
Castelli’s risk index-II	1.65 (0.72–4.61)
AIP	−0.21 (−0.80–0.46)
AC	1.92 (0.83–5.94)
LCI	5.76 (1.27–67.20)
TG/HDL-C ratio	1.39 (0.36–6.66)
METS-IR	40.07 (21.74–68.67)
TyG index	8.26 (7.27–9.52)
TyG-BMI index	239.45 (142.70–372.00)
TyG-WC index	755.74 (481.74–1141.27)

HOMA-IR homeostatic model assessment; HbA1c glycated hemoglobin (A1c); HDL cholesterol high density lipoprotein; LDL cholesterol low density lipoprotein; BMI body mass index; WHR waist to hip ratio; WHtR waist to height ratio; BAI body adiposity index; VAI visceral adiposity index; LAP lipid accumulation product; BRI body roundness index; ABSI a body shape index; AIP Atherogenic Index of Plasma; AC Atherogenic Coefficient; LCI Lipoprotein Combine Index; TG/HDL-C ratio The triglyceride to high-density lipoprotein cholesterol ratio; METS-IR metabolic score for insulin resistance; TyG index Fasting triglycerideglucose index; TyG-BMI index Triglyceride Glucose-Body Mass Index; TyG-WC index Triglyceride Glucose-waist circumference index.

**Table 2:** Correlation between anthropometric parameters, atherogenic indices and glucose profile in the study group.

		N	R	*p*-Value		N	R	*p*-Value		N	R	*p*-Value
Body weight [kg]	Fasting glucose [mmol/L]	49	0.43	0.002	Glucose after 120 min [mmol/L] in glucose tolerance test	40	0.31	0.051	Glucose after 60 min [mmol/L] in glucose tolerance test)	39	0.40	0.011
Waist circumference [cm]		49	0.40	0.004		40	0.37	0.018		39	0.52	0.001
Hip circumference [cm]		49	0.40	0.004		40	0.22	0.172		39	0.29	0.073
BMI index [kg/m^2^]		49	0.43	0.002		40	0.33	0.035		39	0.43	0.006
WHR		49	0.35	0.014		40	0.42	0.007		39	0.65	0.000
WHtR		49	0.37	0.008		40	0.40	0.010		39	0.53	0.000
BAI [%]		49	0.38	0.006		40	0.26	0.099		39	0.26	0.106
VAI		49	0.29	0.042		40	0.44	0.004		39	0.47	0.002
LAP		49	0.38	0.006		40	0.44	0.004		39	0.58	0.000
BRI		49	0.37	0.008		40	0.40	0.010		39	0.53	0.000
ABSI		49	0.15	0.283		40	0.30	0.052		39	0.60	0.000
Castelli’s risk index-I		49	0.33	0.019		40	0.41	0.008		39	0.34	0.031
Castelli’s risk index-II		49	0.33	0.017		40	0.40	0.010		39	0.32	0.047
AIP		49	0.27	0.055		40	0.44	0.004		39	0.46	0.003
AC		49	0.33	0.019		40	0.41	0.008		39	0.34	0.031
LCI		49	0.28	0.046		40	0.50	0.001		39	0.36	0.024
TG/HDL-C ratio		49	0.27	0.055		40	0.44	0.004		39	0.46	0.003
METS-IR		49	0.44	0.002		40	0.35	0.024		39	0.45	0.004
TyG index		49	0.41	0.003		40	0.48	0.002		39	0.54	0.000
TyG-BMI index		49	0.42	0.002		40	0.40	0.009		39	0.48	0.002
TyG-WC index		49	0.42	0.003		40	0.43	0.006		39	0.56	0.000

BMI body mass index; WHR waist to hip ratio; WHtR waist to height ratio; BAI body adiposity index; VAI visceral adiposity index; LAP lipid accumulation product; BRI body roundness index; ABSI a body shape index; AIP Atherogenic Index of Plasma; AC Atherogenic Coefficient; LCI Lipoprotein Combine Index; TG/HDL-C ratio The triglyceride to high-density lipoprotein cholesterol ratio; METS-IR metabolic score for insulin resistance; TyG index Fasting triglycerideglucose index; TyG-BMI index Triglyceride Glucose-Body Mass Index; TyG-WC index Triglyceride Glucose-waist circumference index.

**Table 3:** Correlation between anthropometric parameters, atherogenic indices and insulin profile in the study group.

		N	R	*p*-Value		N	R	*p*-Value
Body weight [kg]	Fasting insulin [pmol/L]	49	0.67	0.000	Insulin after 60 min [pmol/L] in glucose tolerance test	36	0.33	0.046
Waist circumference [cm]		49	0.71	0.000		36	0.39	0.018
Hip circumference [cm]		49	0.56	0.000		36	0.31	0.058
BMI index [kg/m^2^]		49	0.71	0.000		36	0.37	0.023
WHR		49	0.69	0.000		36	0.41	0.013
WHtR		49	0.70	0.000		36	0.40	0.014
BAI [%]		49	0.58	0.000		36	0.32	0.054
VAI		49	0.64	0.000		36	0.30	0.072
LAP		49	0.70	0.000		36	0.36	0.027
BRI		49	0.70	0.000		36	0.40	0.014
ABSI		49	0.40	0.004		36	0.31	0.065
Castelli’s risk index-I		49	0.61	0.000		36	0.19	0.254
Castelli’s risk index-II		49	0.60	0.000		36	0.18	0.282
AIP		49	0.63	0.000		36	0.30	0.074
AC		49	0.61	0.000		36	0.19	0.254
LCI		49	0.58	0.000		36	0.17	0.319
TG/HDL-C ratio		49	0.63	0.000		36	0.30	0.074
METS-IR		49	0.72	0.000		36	0.40	0.015
TyG index		49	0.64	0.000		36	0.34	0.043
TyG-BMI index		49	0.73	0.000		36	0.37	0.025
TyG-WC index		49	0.73	0.000		36	0.41	0.012

BMI body mass index; WHR waist to hip ratio; WHtR waist to height ratio; BAI body adiposity index; VAI visceral adiposity index; LAP lipid accumulation product; BRI body roundness index; ABSI a body shape index; AIP Atherogenic Index of Plasma; AC Atherogenic Coefficient; LCI Lipoprotein Combine Index; TG/HDL-C ratio The triglyceride to high-density lipoprotein cholesterol ratio; METS-IR metabolic score for insulin resistance; TyG index Fasting triglycerideglucose index; TyG-BMI index Triglyceride Glucose-Body Mass Index; TyG-WC index Triglyceride Glucose-waist circumference index.

**Table 4:** Correlations between anthropometric parameters, atherogenic indices and HbA1c and HOMA-IR index in the study group.

		N	R	*p*-Value		N	R	*p*-Value
Body weight [kg]	HbA1c [%]	25	0.20	0.325	HOMA-IR index	49	0.66	0.000
Waist circumference [cm]		25	0.16	0.435		49	0.68	0.000
Hip circumference [cm]		25	0.19	0.346		49	0.57	0.000
BMI index [kg/m^2^]		25	0.13	0.522		49	0.70	0.000
WHR		25	0.13	0.510		49	0.62	0.000
WHtR		25	0.05	0.791		49	0.66	0.000
BAI [%]		25	0.02	0.914		49	0.58	0.000
VAI		25	0.12	0.538		49	0.61	0.000
LAP		25	0.14	0.497		49	0.68	0.000
BRI		25	0.05	0.791		49	0.66	0.000
ABSI		25	0.17	0.394		49	0.33	0.019
Castelli’s risk index-I		25	0.13	0.533		49	0.56	0.000
Castelli’s risk index-II		25	0.19	0.361		49	0.54	0.000
AIP		25	0.10	0.608		49	0.61	0.000
AC		25	0.13	0.533		49	0.56	0.000
LCI		25	0.20	0.335		49	0.54	0.000
TG/HDL-C ratio		25	0.10	0.608		49	0.61	0.000
METS-IR		25	0.11	0.597		49	0.71	0.000
TyG index		25	0.17	0.402		49	0.63	0.000
TyG-BMI index		25	0.13	0.536		49	0.72	0.000
TyG-WC index		25	0.24	0.248		49	0.71	0.000

HOMA-IR homeostatic model assessment; HbA1c glycated hemoglobin (A1c); BMI body mass index; WHR waist to hip ratio; WHtR waist to height ratio; BAI body adiposity index; VAI visceral adiposity index; LAP lipid accumulation product; BRI body roundness index; ABSI a body shape index; AIP Atherogenic Index of Plasma; AC Atherogenic Coefficient; LCI Lipoprotein Combine Index; TG/HDL-C ratio The triglyceride to high-density lipoprotein cholesterol ratio; METS-IR metabolic score for insulin resistance; TyG index Fasting triglycerideglucose index; TyG-BMI index Triglyceride Glucose-Body Mass Index; TyG-WC index Triglyceride Glucose-waist circumference index.

## Data Availability

All date are availability by address email of justyna.nowak@sum.edu.pl. Data is not publicly available due to privacy or ethical restrictions.
